# A hybrid deep learning–based OCR model for handwritten medical prescriptions

**DOI:** 10.3389/fmed.2026.1856485

**Published:** 2026-10-05

**Authors:** Omar Z. Ameer, Mahmood Hameed Majeed, Safa L. Kailan, Saif Mohamed Baraa Alsabti, Ali M. Elhenidy

**Affiliations:** 1 College of Pharmacy, Alfaisal University, Riyadh, Saudi Arabia; 2 Department of Biomedical Engineering, College of Engineering, Al-Nahrain University, Baghdad, Iraq; 3 Technical College of Engineering, Al-Bayan University, Baghdad, Iraq; 4 Computer Engineering and Control Systems Department, Faculty of Engineering, Mansoura University, Mansoura, Egypt

**Keywords:** GhostNetV2, healthcare automation, LLM, medical prescription digitization, optical character recognition

## Abstract

The current trend involving the use of paper-based medical prescriptions and the exchange of such by the social media is a major problem in automated and accurate text extraction in healthcare processes. This paper suggests a complete smart pipeline to digitize the images of medical prescriptions with lightweight deep learning-based classification, specialized Optical Character Recognition (OCR) engines, and comparative benchmarking of results using a large language model (LLM). The web scraped images of medical prescriptions are initially processed through a GhostNetV2 classifier to differentiate between handwritten and digital medical prescriptions. According to this choice, digital images are sent to PaddleOCR, and handwritten images are considered in Tesseract OCR so that each image could be processed by the best suitable recognition engine. The OCR outputs from both branches are benchmarked against GPT-4o (OpenAI) as an independent baseline to assess competitive performance and provide error-rate context. Experimental analysis shows that the suggested adaptive routing strategy is characterized by the fact that it has a high classification rate and the character and word error rates are significantly lower than those single-OCR techniques. The system is competitive with, though does not exceed, the raw accuracy of a state-of-the-art LLM-based OCR baseline (GPT-4o), while offering practical advantages in cost, transparency, and on-premises deployability that the black-box, per-call LLM baseline does not. The findings verify that the combination of lightweight classifiers and multi-OCR decision routing and multi-LLM-aided comparative benchmarking offers a scalable and robust solution to real-world medical prescriptions digitization, which adds to better automation, accuracy, and safety in healthcare information processing.

## 1 Introduction

The healthcare sector continues to rely largely on paper-based prescriptions, which are often shared informally through social media platforms such as WhatsApp and Messenger. Since the age of agentic AI and automation has come allowing it to convert handwritten and printed prescription images to machine-readable text is now possible. One of the promising opportunities is the ease of Optical Character Recognition (OCR) technology that enhances the accuracy and efficiency of the healthcare processes.

The method (1) based on neural networks enhances the OCR accuracy of faxed medications to a large extent. The complex pre-processing strategies, multi-modal segmentation strategies and combination of learned and manually developed features. A digital copy of handwritten prescriptions is created with the help of an AI-powered optical character recognition (OCR) system (2) based on Roboflow and Transformer-based Optical Character Recognition (TrOCR). Text digitization of images is the industry standard, which is optical character recognition, or OCR. As an example, Tesseract is utilized in the work in (3) to recognize information in photos of handwritten and printed prescriptions. It also compares specific fields such as prescription names, dosages, and patient directions through the data extraction of the correct format by using regular expression. Similarly, the article in (4) involves the use of OCR to extract the data and recommends two methods of handling the handwritten and printed prescriptions, respectively: the 3-step filtering algorithm and C-cube algorithm. The aim of the proposed algorithm was to form clusters based on which a relevant data could be processed. Other researchers (5) are willing to employ classical machine learning models such as the Logistic regression, and the Support Vector Machines deployed in conjunction with data augmentation to improve the quality of the images submitted to the OCR model which is Tesseract in the given case. The said methodology was utilized to categorize Medical and Non-Medical scans. The other trend is the use of the pretrained Large Language Models to retrieve the text of the visual images. One of this To identify meaningful data within medical prescriptions, such as medication dosage and frequency the OpenAI GPT-4 model was adopted in (6) to convert the visual data into text data frames to further analyze them. A recent article (7) has suggested a lightweight workflow based on the Optical Character Recognition (OCR) and MobileNetV2 (8) to read and classify images respectively. The extracted text is processed by the Rapid Automatic Keyword Extraction (RAKE) algorithm so that to identify important keywords. Then, a Google Search API is employed to be able to match these keywords to relevant URLs. The new, AI-driven solution of the HandIntellect AI (9) changes the ineffective handwritten notes into the clear and digital language. It utilizes a massive language model (LLM) to facilitate more precise information understanding and Tesseract.js to read characters (OCR).

PresCa (10) offer such valuable features as prescription scanning, scanning recommendations, report summary, a support Relational Agent, prescription crosscheck by doctors, and the possibility to set up medicine. In this paper, the author will show how to retrieve a pharmaceutical information on handwritten prescriptions with the help of a simple deep learning model known as TrOCR: Transformer-Based Optical Character Recognition. This pre-trained model can be used to identify complex handwritten language reliably by using the Optical Character Recognition (OCR) technique and the Transformer architecture. Moreover, the speed and accuracy of prescription scanning can be improved by including the use of the technologies of Machine Learning (ML) in the app, as it helps users be able to access and understand their medication instructions.

Although OCR-based prescription-digitization has been extensively studied, there is a research gap to be filled in end-to-end adaptive OCR pipelines which explicitly consider the heterogeneity of source and writing style of prescription before text extraction. The majority of the available ways are based on enhancing OCR accuracy either by using preprocessing or segmentation or Transformer-based recognition or using one OCR engine to process both printed and handwritten prescriptions. Although there are also works that suggest classification or filtering approaches, they usually focus on the major divisions like medical and non-medical documents, but not narrow ones like handwritten and digital prescription. Furthermore, recent LLM based methods usually do direct image-to-text extraction with no prior decision step in the process of the best OCR engine, which may result in inconsistent performance with different prescription types and actual image characteristics that can be found in social-media-shared prescriptions.

The gap is bridged by the suggested work which suggests an adaptive, classifier-based OCR routing model that is customized according to the requirements of the real-life scenario which entails the obtaining of medical prescription images by the use of web scrapbots and social networks. Unlike the earlier study which utilizes a single OCR model or solely employs LLMs to access information, the current study integrates a model of a low-weight GhostNetV2 classifier as a front-end decision-making unit, which allows gaining a better insight into the resemblance between handwritten and digital prescriptions. By designing this system in this manner, the system is able to pass dynamically over digital images to PaddleOCR and handwritten images to Tesseract to take advantage of the capabilities of both OCR engines. In this study, a medical prescription is defined as a document issued by a licensed healthcare professional that authorizes the dispensing of one or more medications and specifies the associated dosage and administration instructions. The dataset used in this work also contains healthcare-related pharmaceutical images, such as medication labels and packaging, which are treated as a separate category because they frequently coexist with prescription documents in practical pharmacy workflows. Accordingly, the scope of this work is the processing of heterogeneous prescription-related healthcare imagery rather than exclusively prescription documents.

The method suggested has based classification and recognition independent of each other, and this encourages reduction of cross-domain OCR errors and a better robustness to various prescription forms, handwriting and image acquisition characteristics. The key novelty of this work is three-fold: (1) an adaptive, classifier-guided OCR routing strategy using GhostNetV2 that explicitly separates handwritten and digital prescriptions before OCR processing, unlike prior work that applies a single engine to all inputs; (2) a dual-engine OCR architecture combining PaddleOCR for digital text and Tesseract for handwritten content, specifically calibrated for the heterogeneity of real-world prescription images collected from social media and web sources; and (3) an LLM-based comparative benchmarking layer using OpenAI OCR as a secondary quality-assurance mechanism, which departs from existing pipelines that either use LLMs as a sole extractor or forgo post-OCR comparative benchmarking entirely. This end-to-end design explicitly addresses real challenges specific to social-media-shared prescription images including variable image quality, diverse handwriting styles, inconsistent layouts, and noise introduced by compression artifacts. The synergy of lightweight CNN-based classification, specialized multi-engine OCR routing, and LLM-aided comparative benchmarking constitutes the key contribution of the given paper. It is a hybrid architecture that integrates a lightweight CNN classifier, specialized OCR engines, and large language models within a single pipeline. This offers a scalable and adaptable solution to the demands of automated, precise, and intelligent prescription digitization in modern healthcare systems. Separately from these three validated contributions, the pipeline also includes a preliminary medicine-name identification step, illustrated on the extracted text from digital prescriptions using lexicon-based keyword matching to flag candidate drug names; this step is exploratory only, has not been quantitatively evaluated (e.g., no precision/recall against annotated drug mentions), and is therefore not counted among the paper's contributions. The rest of the paper is organized as follows: Section 2 discusses the dataset and preprocessing techniques. Section 3 illustrates the proposed model. Results and discussion are presented in Section 4 and the conclusion is in Section 5.

## 2 Dataset

Consistent with the definition adopted in the Introduction, a medical prescription is defined in this study as a document issued by a licensed healthcare professional that authorizes the dispensing of one or more medications and specifies the associated dosage and administration instructions. Medication labels and packaging images are not treated as prescriptions under this definition; they are retained as a separate, explicitly distinguished category of healthcare-related pharmaceutical imagery, reflecting the heterogeneous content encountered in real-world social-media-shared prescription images. The dataset was gathered with the aid of web crawling and real mobile camera shots. Images were manually reviewed and divided into two groups: handwritten images (physician-written notes and prescriptions) and digital images (printed prescriptions, pharmaceutical product labels, and related medical product imagery). The digital class includes a range of printed pharmaceutical content rather than exclusively formatted e-prescriptions, and this is acknowledged as a scope limitation. The overall dataset comprises 508 images: 175 handwritten and 333 digital.

Unlike conventional prescription datasets that contain only prescription forms, the proposed dataset includes both prescription documents and healthcare-related pharmaceutical images. These categories are explicitly distinguished throughout this work. The inclusion of pharmaceutical imagery was intended to improve the robustness of the framework under realistic pharmacy scenarios; however, these images are not considered medical prescriptions, and the manuscript has been revised accordingly.

### 2.1 Preprocessing

The data is obtained through the combination of mobile camera shots and the images obtained via the Internet. This compromise character presents problems of image resolution, angle and consistency. Therefore, there is a need to have a preprocessing phase to manage this problem. The list of the preprocessing techniques will be listed in this section.

### 2.2 Dataset challenges

The collected dataset presents a number of real-world challenges that must be explicitly addressed to ensure robust pipeline performance. First, for web-scraped digital prescription images, the primary challenge is the heterogeneity of layouts, fonts, and languages across different prescription formats. Scraped images often lack consistent resolution, may contain watermarks, logos, or stamps, and can include both printed and handwritten annotations on the same document. To verify the presence of medicine names in web-scraped images, the OCR-extracted text was matched against a standardized drug name lexicon (derived from WHO essential medicines and national formularies), and matching entities were flagged as medicine candidates. This lexicon-based post-processing step confirmed that medicine names were identifiable in the digital prescription images and provided a structured output for downstream clinical comparative benchmarking. Second, for handwritten prescription images, the challenges are significantly more severe. Handwriting style varies considerably across physicians, with strokes ranging from neat print-style to highly cursive and abbreviated forms. Additional factors such as smudging, low lighting conditions during capture via mobile camera, skewed scanning angles, and domain-specific abbreviations (e.g., “sig”, “qid”, “prn”) further degrade OCR accuracy. The adaptive routing strategy employed in this pipeline directly addresses these challenges by ensuring that the specialized Tesseract engine, pre-configured for handwritten text, receives only handwritten prescription images rather than being applied indiscriminately. The preprocessing steps described in subsequent sections are designed to mitigate these image-quality degradation factors before any OCR processing occurs.

Although the proposed dataset contains a substantial number of prescription images, it also includes healthcare-related pharmaceutical images such as medication labels and packaging. These images were included to better represent practical pharmacy environments; however, they are not equivalent to prescription documents. Consequently, the conclusions of this study should be interpreted within the context of heterogeneous prescription-related healthcare imagery. Future work will focus on expanding the proportion of authentic prescription documents collected through collaborations with healthcare providers and pharmacies.

### 2.3 Image resizing

To make the input dimensions uniform, all the images are resized to a constant resolution (512  ×  512 pixels). In scaling an image, the adding of padding that is adding of borders to an original image assists it to maintain its aspect ratio. This prevents distortion of non square pictures during resizing.

### 2.4 Normalization

The normalization has the benefit of speeding up convergence of training and reducing the effect of varying image intensities. Standardization or (Z-Score Normalization) transfigures data such that it has a mean of 0 and a unit variance as shown in equation 1.

$$I_{normalized}=\;\frac{I-\;\mu }{\sigma }$$ (1)

Where:

I: is the original image’s pixel intensity.

*σ*: is the standard deviation of the pixel intensities.

*μ*: is the mean of the pixel intensities.

### 2.5 Data augmentation

In order to augment the dataset, a number of augmentation methods are used, including picture rotation, translation, scale, shear, flip left-right, and flip up-down. These techniques of geometric transformations decrease the difference in orientation or angle. To enhance the generalization and strength of the models, another data augmentation technique known as CutMix (14) is embraced. The success of integrating the content and supervision cues of two pictures and the labels associated with them enhances training. Based on the data enhancement of CutMix, mosaic data enhancement randomly scales, crops, and orders four photos to form another image (14). Enhancing mosaic data can reduce its cost, add value, enhance the resilience of the network, and require fewer graphics processing units (GPUs). The CutMix mathematical equation is shown in equations 2 and 3. An example of this augmentation technique applied during training is shown in Figure 1 (pipeline diagram).

$$x_{new}=M⊙\;x_{A}+(1-M)⊙\;xB$$ (2)

$$y_{new}=\;\lambda \;y_{A}\;+(1-\;\lambda \;)\;yB$$ (3)

**Figure 1:**
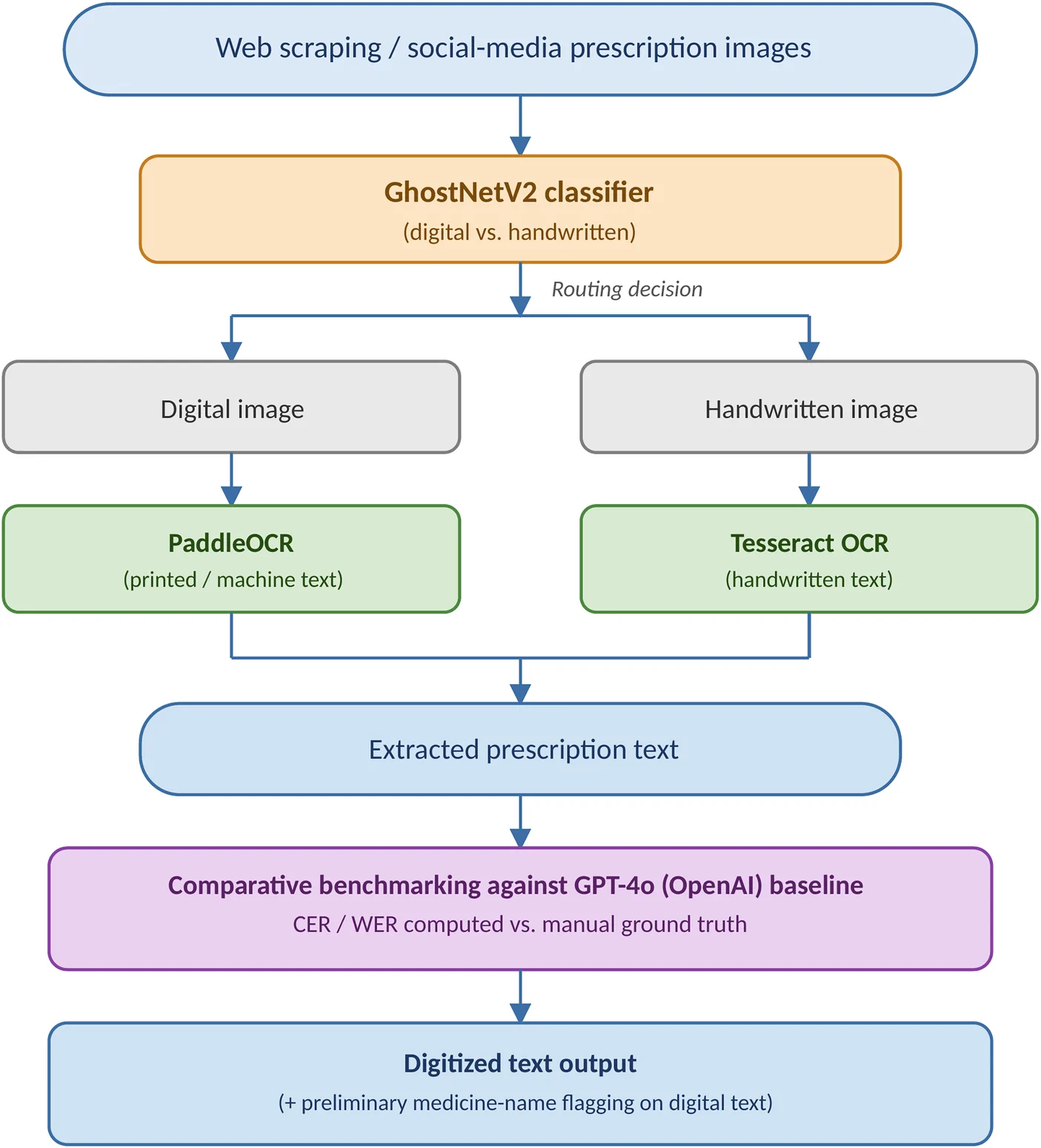
The proposed line.

Where:

M: is a conversion of the patch area in binary

⊙: is element-wise multiplication.

$$\lambda =\frac{\;patch\;area}{image\;area}$$

$x_{A}$ and $y_{A}$ are the image and label for the first sample.

xB and yB are the image and label for the second sample.

## 3 The proposed pipeline

As illustrated in Figure 1, the suggested pipeline -offers an end-to-end automated system to process medical prescription images, beginning with data acquisition and concluding with strong text comparative benchmarking. The former phase is based on web scraping to gather images of medical prescriptions in online sources. This move allows massive, real-world data gathering, includes a variety of prescription formats, resolutions, and noise which can mirror real deployment situations. The images that have been scraped are then processed into medical prescription inputs in a way that forms the basis of the further intelligent processing levels.

After gathering, the medical prescription images are forwarded to the GhostNetV2 classifier, which serves as a lightweight and accurate front-end decision module. Given frequent similarity in the intermediate feature maps of the standard convolutional network, GhostNet (11) is proposed to propose a smaller number of the actually necessary convolution filters generating them. The ghost module eliminates the bias term in the convolution calculations and uses the linear computations in order to compute the intermediate features. A series of linear operations are used to compute intrinsic features as illustrated in Figure 2. Ghost module demonstrates its effectiveness in the tasks of pedestrian detection on the basis of YOLO architecture (12, 13).

**Figure 2:**
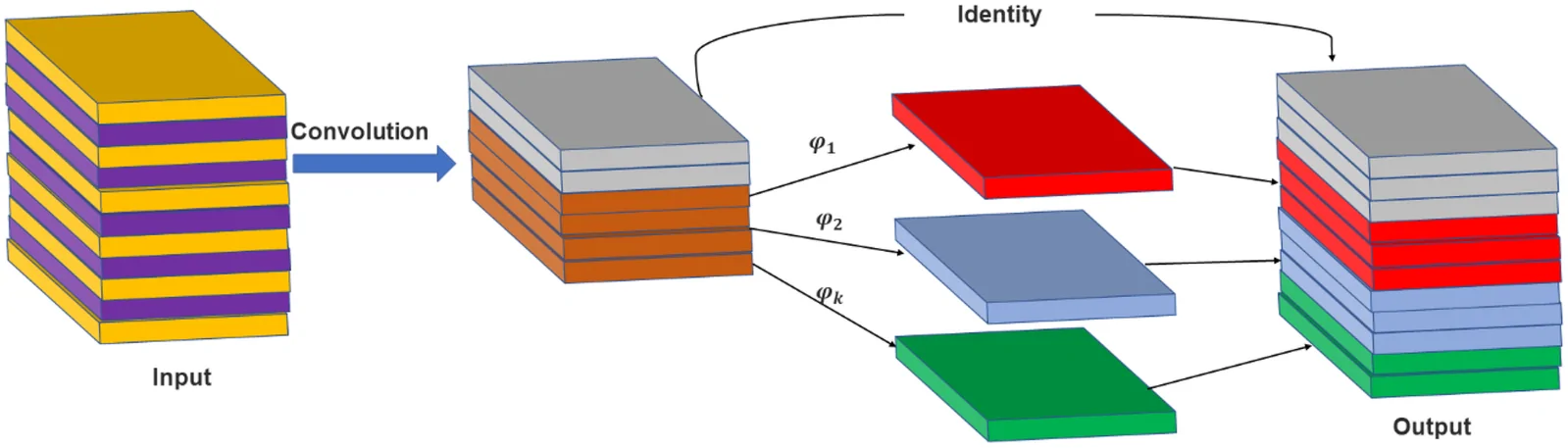
Ghost module.

GhostNetV2 was selected for its favorable efficiency profile: with approximately 3.9 million parameters and around 0.17 GFLOPs per forward pass, it achieves a strong accuracy/efficiency trade-off relative to alternatives such as MobileNetV2 (3.5M parameters, similar accuracy) and EfficientNet-B0 (5.3M parameters). Inference latency on the RTX 2060 is approximately 7.5 ms per image (Table 1), making it suitable for real-time deployment in clinical or pharmacy settings. GhostNetV2 uses visual qualities of each image, including font uniformity, stroke irregularities, and layout patterns, to classify the prescription as either digital or handwritten. This classification stage acts as a routing gate, ensuring that each image is directed to the OCR engine most appropriate for its content type, thereby improving overall recognition accuracy and efficiency.

**Table 1:** Comparison of lightweight CNN backbones.

Model	Parameters (M)	FLOPs (G)	Latency (ms/image)	Accuracy (%)	Precision (%)	Recall (%)	F1-score (%)
ShuffleNetV2	2.3	0.15	7.8	98.2	98.0	98.3	98.1
MobileNetV3-Small	2.5	0.07	7.1	98.6	98.5	98.6	98.5
MobileNetV2	3.5	0.30	8.9	98.8	98.7	98.8	98.7
EfficientNet-B0	5.3	0.39	12.4	99.0	98.9	99.0	98.9
GhostNetV2 (Proposed)	3.9	0.17	7.5	99.0	99.0	99.0	99.0

When GhostNetV2 determines an image to be digital, it is passed on to PaddleOCR that is specialized in printed machine text with structured layouts and uniform typography. PaddleOCR is a highly precise text extraction behavior of digital prescriptions with proven clean and structured text outputs. The proposed branch of the pipeline will achieve the maximum level of accuracy on the medical documents that are to be printed, including e-prescriptions and product labels. In the vice versa case, when the image is considered to be handwritten, it is sent to Tesseract OCR that is more appropriate in dealing with non-regular handwriting and non-regular character shapes. Tesseract is used to handle such handwritten prescriptions to induce textual information that would otherwise have been difficult with regular OCR systems. The pipeline reduces the cross-domain error by isolating handwritten to digital input which increases recognition strength of complex handwritten medical notes.

In the last phase, the text of both OCR streams found in the PaddleOCR and Tesseract are compared with the text obtained as a result of the OCR application of OpenAI strength. The step of comparison serves as a benchmarking mechanism: CER and WER between the pipeline output and the GPT-4o output are computed to flag cases of high divergence for human review. Both systems are evaluated against the same manually annotated ground truth to ensure that the comparison is meaningful. GPT-4o (model gpt-4o-2024-05-13) was accessed via the OpenAI API with the prompt: “Extract all text from this medical prescription image exactly as written, preserving line breaks.” It is presented as an independent baseline rather than a gold standard, given that its own output is subject to hallucination and model version changes.

## 4 Simulation analysis and results

### 4.1 Ground-Truth annotation procedure

To ensure a fair and reproducible evaluation of the proposed OCR framework, a manually verified ground-truth dataset was established for all prescription and healthcare-related images used in the OCR experiments. The ground-truth transcriptions were independently prepared by two annotators with experience in reading handwritten and digital medical prescriptions. Each annotator manually transcribed the complete textual content of every image while preserving the original wording, line order, abbreviations, numerical values, and punctuation whenever applicable. The annotation process followed a predefined protocol to ensure consistency across all samples.

After the independent transcription stage, the annotations produced by the two annotators were compared. Whenever discrepancies were identified, including differences in character recognition, word spelling, abbreviations, or formatting, the corresponding samples were independently reviewed by a third reviewer with experience in prescription interpretation. The third reviewer adjudicated the disagreements, and a consensus transcription was established for each image. The resulting consensus transcription served as the gold-standard reference throughout this study. All OCR methods evaluated in this work, including PaddleOCR, Tesseract OCR, and the OpenAI-based OCR baseline, were assessed against this common reference. The Character Error Rate (CER) and Word Error Rate (WER) reported in the manuscript were computed exclusively by comparing the OCR outputs with these manually verified ground-truth transcriptions.

It is important to note that the OpenAI-based OCR model was not used to generate or verify the ground-truth annotations. Instead, it was evaluated as an independent baseline under the same experimental conditions as the other OCR engines. Consequently, all reported OCR performance metrics were obtained using a common manually established reference, ensuring a fair and unbiased comparison among all evaluated methods.

### 4.2 Evaluation metric

The performance indicators of the models are their recall and precision. Equation (4) describes precision as the ratio of samples correctly classified as belonging to a given class (true positives) to the total number of samples predicted as that class. The ratio of samples correctly classified as belonging to a given class to the total number of samples that actually belong to that class is called the recall, which is depicted in equation (5). The F1 Score applies the algorithm to balance these measurements as expressed in equation (6). The values of false positives, false negatives, and true positives have a significant effect on the F1 Score. Increase in false negatives would decrease recall and increase in false positives would decrease precision leading to a lower F1 Score. Conversely, memory and precision are enhanced by enhancing the true positives, and the F1 Score is raised. Accuracy as mentioned in equation (7) measures the overall correctness of a classification model by calculating the proportion of all predictions—both positive and negative—that the model correctly identifies. It is computed as the ratio of correctly classified instances (true positives and true negatives) to the total number of samples.

Character Error Rate (CER) is the primary metric used to evaluate the accuracy of AI models that transcribe or recognize text, such as Optical Character Recognition (OCR), handwritten text recognition (HTR), and speech-to-text systems. It measures the percentage of characters a model gets wrong. Character Error Rate (CER) equation is listed in Equation (8). Word Error Rate (WER) as listed in Equation (9) is the standard metric used to evaluate the accuracy of speech-to-text (STT) and machine translation systems. It calculates the percentage of words a system incorrectly transcribes, deletes, or adds compared to a perfect human reference transcript.

$$Precison\;=\frac{TP}{TP+FP}$$ (4)

$$Recall\;=\frac{TP}{TP+FN}$$ (5)

$$F1=\frac{2*precision*recall}{\;precision+recall}$$ (6)

$$Accuracy\;=\frac{TP+TN}{TP+\mathrm{TN}+FN+FP}$$ (7)

$$CER\;=\frac{S+D+I}{N}\;\times 100$$ (8)

$$WER\;=\frac{S+D+I}{Nw}\;\times 100\;$$ (9)

Where:

- S: Number of Substitutions (wrong characters in place of correct ones)
- D: Number of Deletions (missing characters)
- I: Number of Insertions (extra characters added)
- N: Total number of characters in the reference text (used in the CER formula, equation 8)
- Nw: Total number of words in the reference text (used in the WER formula, equation 9). For WER, S, D, and I likewise count substituted, deleted, and inserted words rather than characters.

These classification metrics are combined with OCR error rates to give a comprehensive evaluation of both pipeline stages.

These metrics are used to evaluate the binary classification performance of the GhostNetV2 routing stage, as well as to assess OCR quality via Character Error Rate (CER) and Word Error Rate (WER) computed against manually produced ground-truth transcriptions. CER measures the proportion of incorrectly recognized characters, while WER measures the proportion of incorrectly recognized words, both relative to the reference transcription.

### 4.3 Specification for experiments

The experiments were carried out on a system running Windows 11 with an Intel Core i7 processor and an NVIDIA RTX 2060 GPU (6 GB VRAM). The deep learning framework used is PyTorch 2.0. All prescription images were resized to 512 × 512 pixels prior to classification, consistent with the preprocessing described in Section 2.3. The GhostNetV2 binary classifier was trained from scratch (without pre-trained weights) for 50 epochs using the Adam optimizer with a learning rate of 1 × 10⁻^3^ and a batch size of 32. An 80/20 train/test split was applied to the 508-image dataset (406 training, 102 test). For Tesseract OCR (v5.3.0), the LSTM engine mode (OEM 1) and automatic page segmentation mode (PSM 3) were used, with English and Arabic language data loaded (eng + ara) to handle the mixed-script content present in the dataset. For PaddleOCR (v2.7), the PP-OCRv4 detection and recognition models were used with angle classification enabled and the language set to English only. Unlike Tesseract, which was configured for English and Arabic, PaddleOCR's English-only configuration means that any Arabic text present in digital prescription images is not correctly recognized by the digital branch of the pipeline; this is a limitation of the current implementation, and the reported digital-branch CER/WER should be interpreted as an upper bound that would likely improve with a bilingual PaddleOCR configuration. Neither OCR engine was fine-tuned; both were run with their default pre-trained models under the configurations described above. The OpenAI baseline used the GPT-4o model (accessed via the OpenAI API, model string gpt-4o-2024-05-13) with the prompt: “Extract all text from this medical prescription image exactly as written, preserving line breaks.” All systems were evaluated against manually produced ground-truth transcriptions prepared by two independent annotators with disagreements resolved by a third reviewer.

### 4.4 Experimental results on medical prescription dataset

The GhostNetV2 model was trained on the medical prescription dataset for 50 epochs to classify images as digital or handwritten. The classification report in Table 2 indicates that the GhostNetV2 model successfully distinguishes between digital and handwritten medical prescription images. For the digital class, the precision is 0.98 and the recall is 1.00, meaning all digital images in the test set were correctly identified with virtually zero false positives. This implies that graphical characteristics of digitally produced prescriptions, such as homogenized fonts, uniformity on alignments and clean backgrounds are highly captured in the learnt feature representations of the model.

**Table 2:** The classification report.

Class	Precision	Recall	F1-score	Support
Digital	0.98	1.00	0.99	61
Handwritten	1.00	0.98	0.99	41
Accuracy	-	-	0.99	102
Macro Avg	0.99	0.99	0.99	102
Weighted Avg	0.99	0.99	0.99	102

The classifier in the case of handwritten has a precision of 1.00 and a recall of 0.98 that provides us an F1-score of 0.99. This demonstrates that nearly all the handwritten specimens were identified as well as that only a very small fraction of them had been misidentified as digital. The perfect accuracy means that in the instances where the model represents a particular image to be handwritten, then the model is highly accurate. This is especially true of the OCR pipeline where handwritten images are passed to an expert recognition engine and any misclassification may majorly reduce the quality of the text extraction. Overall, the system has a precision of 99 and macro as well as weighted averages of 0.99 which confirms that there is a balanced and consistent performance between both classes despite the imbalance in the dataset (more digital than handwritten images). These results prove that GhostNetV2 is a useful and efficient front-end classifier that can easily be applied in the field of the real-life case since to route medical prescription images to corresponding OCR models is necessary, and GhostNetV2 is fast and efficient to make such decisions.

As shown in the confusion matrix of Figure 3, the GhostNetV2 classifier is very robust in its ability to differentiate between digital and handwritten medical prescription images. The digital class had a perfect recall since all the digital images in the test set were correctly recognized as such and none as the handwritten ones. This finding shows how the model is strong in determining machine-printed text patterns, layouts, and visual consistency that are usually inherent to digital prescriptions and product images.

**Figure 3:**
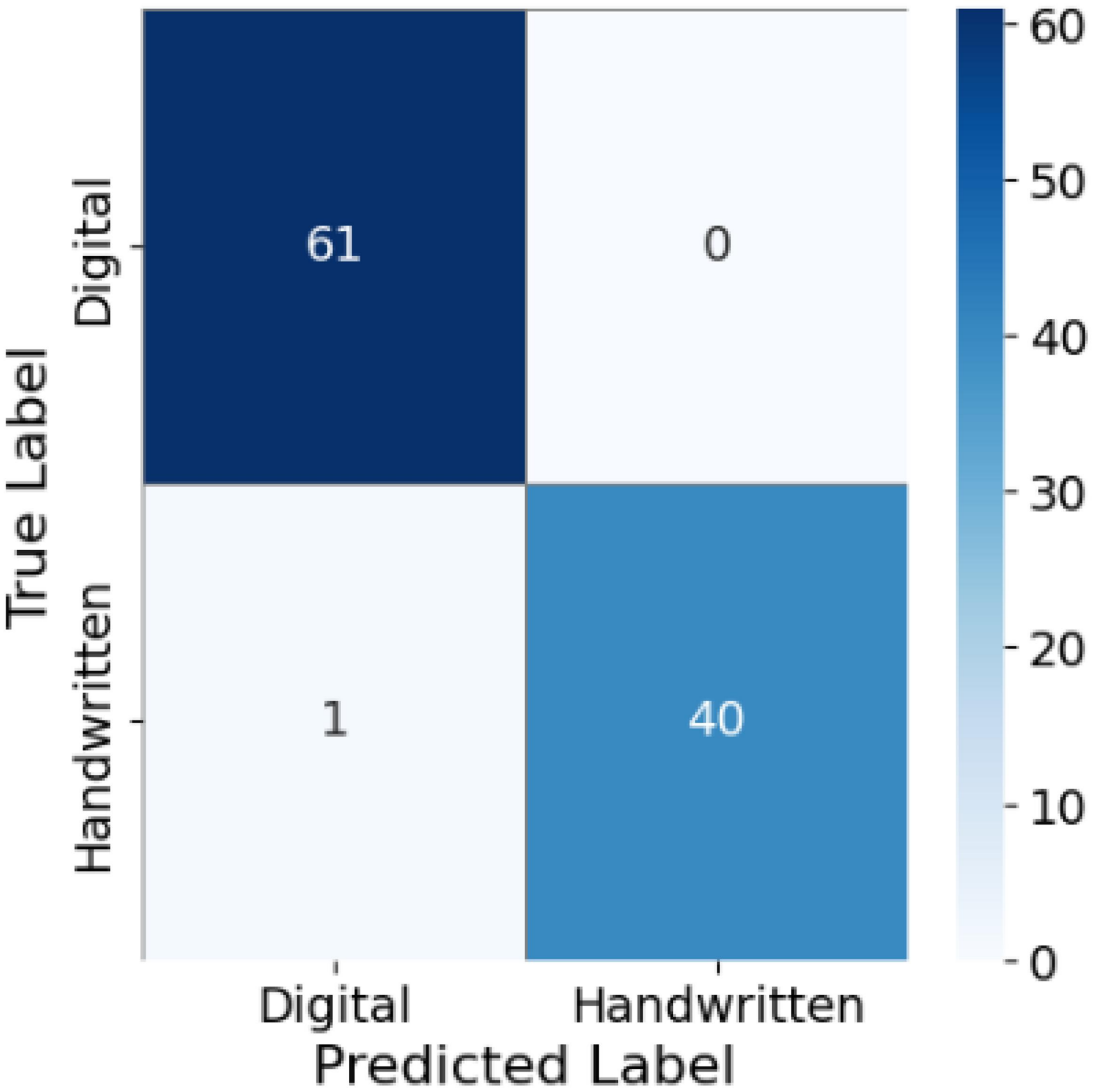
The confusion matrix.

In the case of the handwritten, the model had an accuracy of 40 handwritten images and only one misclassified handwritten image was owned by the model as a digital image. This minor misunderstanding implies that even though handwritten samples can sometimes have visual similarities with electronic text (e.g., neat handwriting or even homogeneous backgrounds), the classifier does not lose high discrimination rates. All in all, confusion matrix indicates high performance, low misclassification, and balanced performance, thus GhostNetV2 is a trusted front-end network classifier to route images to the suitable OCR engine (PaddleOCR to process digital text and Tesseract to process handwritten text) in your suggested medical prescription processing pipeline.

The ROC curve in Figure 4 shows that the GhostNetV2 classifier has excellent discriminative ability on both digital and handwritten image classes. The two curves of the handwritten and the micro-average are very much near the top-left corner of the plot implying the highest true positive rate at very low false positive rates implying an area under the curve (AUC) that is very near to 1.0. This is a positive indication of the confidence and consistency of the model in the separation of handwritten pictures and digital images. The curve of digital class, in contrast, still remains a good performance curve but at a slower rate of increase, indicating that it is slightly more sensitive to false positives. On the whole, the ROC analysis proves that the proposed classifier is very reliable and suitable as a decision-making step in directing medical prescription images to the relevant OCR pipeline.

**Figure 4:**
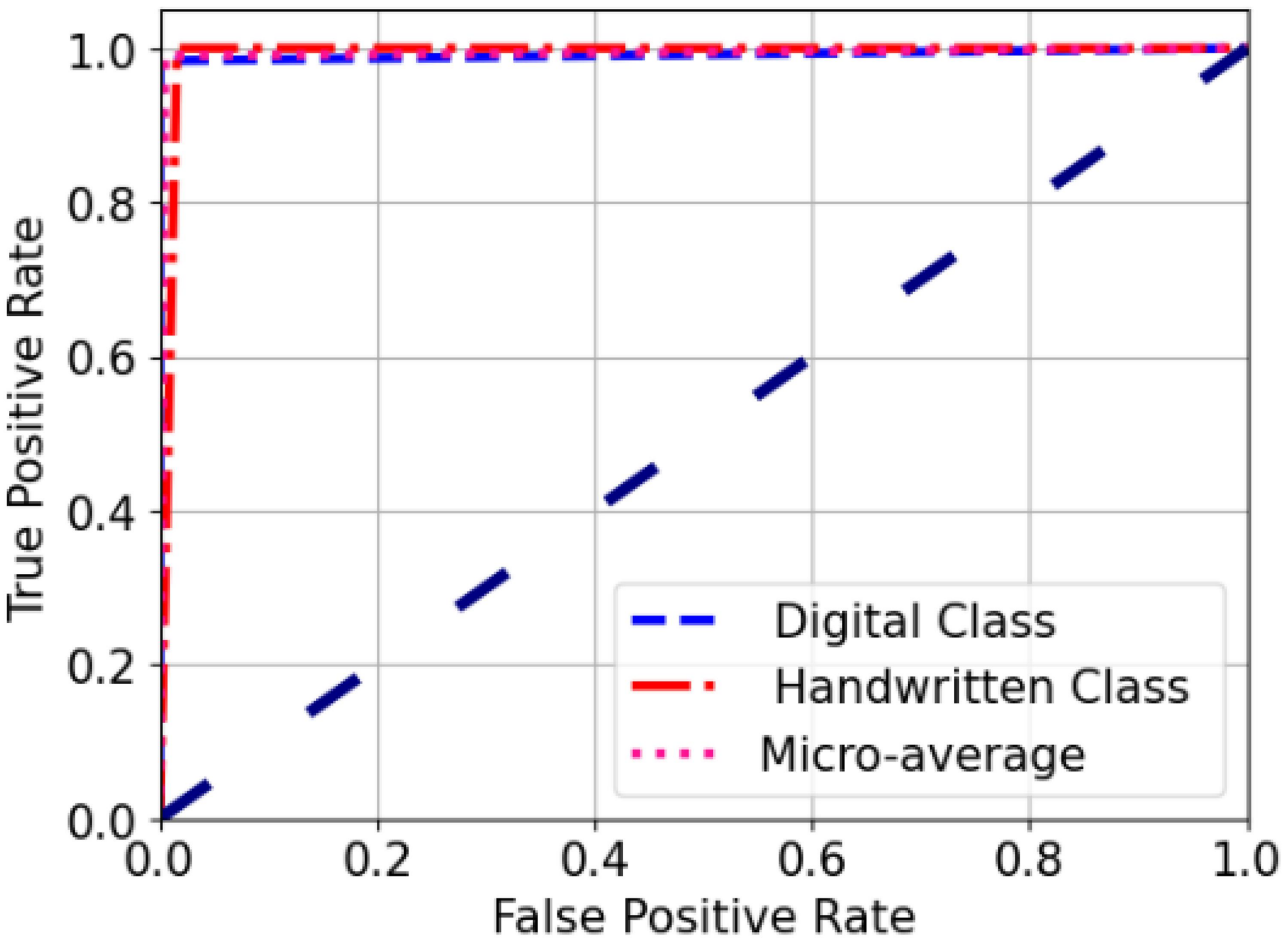
The ROC curve.

#### 4.4.1 Cross-validation robustness check

To directly test whether the 99.0% accuracy reported in Table 2 depends on the specific 80/20 split used, we additionally performed 5-fold stratified cross-validation over the complete 508-image dataset, retraining GhostNetV2 from scratch for 50 epochs on each fold under the same protocol described in Section 4.3 (Adam optimizer, learning rate 1 × 10⁻^3^, batch size 32, 512 × 512 input resolution). Table 3 reports the per-fold and aggregated results.

**Table 3:** 5-Fold stratified cross-validation results (GhostNetV2 classifier).

Fold	N (train/val)	Accuracy (%)	Precision (%)	Recall (%)	F1-score (%)
1	406/102	94.12	94.11	94.12	94.07
2	406/102	90.20	90.44	90.20	89.95
3	406/102	97.06	97.29	97.06	97.09
4	407/101	94.06	94.50	94.06	94.13
5	407/101	90.10	90.35	90.10	89.85
Mean ± SD	–	93.11 ± 2.96	93.34 ± 2.96	93.11 ± 2.96	93.02 ± 3.10

The cross-validated accuracy is 93.11% ± 2.96% (mean ± standard deviation across the five folds; weighted precision 93.34% ± 2.96%, weighted recall 93.11% ± 2.96%, weighted F1-score 93.02% ± 3.10%), with per-fold accuracy ranging from 90.10% to 97.06%. This is materially lower than the 99.0% accuracy obtained on the original single split (Table 2), confirming the reviewer's concern that the single-split estimate was optimistic rather than representative: classification performance is meaningfully split-dependent at the current dataset size, particularly for the smaller handwritten class (33–35 validation images per fold). We therefore treat 93.1% ± 3.0% weighted accuracy as the more reliable estimate of GhostNetV2's classification performance going forward, and retain the original single-split breakdown in Table 2 only for continuity with the confusion-matrix and ROC analyses in Figures 3, 4, which were computed on that split. Because the backbone comparison in Table 1 and the efficiency analysis in Table 4 were likewise obtained on a single 80/20 split, the same fold-sensitivity demonstrated here should be assumed to apply to those comparisons as well; extending 5-fold cross-validation to all backbones in Table 1 is left for future work. Growing the dataset, in particular the handwritten class, remains an important direction to further stabilize this estimate.

**Table 4:** Efficiency trade-off analysis.

Model	Accuracy (%)	FLOPs (G)	Parameters (M)	Efficiency Score (Accuracy/FLOPs)
ShuffleNetV2	98.2	0.15	2.3	654.7
MobileNetV3-Small	98.6	0.07	2.5	1408.6
MobileNetV2	98.8	0.30	3.5	329.3
EfficientNet-B0	99.0	0.39	5.3	253.8
GhostNetV2 (Proposed)	99.0	0.17	3.9	582.4

The standard classification and OCR assessment measures were used to evaluate the proposed pipeline on the gathered medical prescription and pharmaceutical-imagery dataset. As noted in Section 2.1, the “digital” class is not composed exclusively of prescription documents: it also includes pharmaceutical product labels and packaging images, which are explicitly not medical prescriptions under the definition adopted in this study. Consequently, the CER/WER figures reported below characterize the pipeline's text-extraction performance across this broader, heterogeneous set of prescription-related healthcare imagery, and should not be read as prescription-only digitization performance; we use the qualifier “prescription and pharmaceutical-image digitization” for this reason. Disaggregating OCR performance by prescription-only vs. label/packaging-only sub-classes is left for future work, since the current ground-truth annotation does not separate these two sub-categories within the digital class. In the classification step, GhostNetV2 was found to have an accuracy of 99.0%, with a weighted precision of 0.99, recall of 0.99, and F1-score of 0.99 on the original single 80/20 split (Table 2); as detailed in Section 4.4.1, 5-fold cross-validation gives a more robust estimate of 93.1% ± 3.0% weighted accuracy, which we treat as the representative performance figure for this classifier going forward, while the single-split figures above are retained for continuity with the confusion-matrix and ROC analyses. The adaptive routing strategy achieved a Character Error Rate (CER) of 4.1% and Word Error Rate (WER) of 7.6% on the entire dataset at the OCR level. Specifically, PaddleOCR achieved a CER of 3.2% on digital (machine-printed) prescriptions, while Tesseract achieved a CER of 5.0% on handwritten prescriptions. It is important to note that handwritten medical prescription recognition is inherently more challenging than digital OCR, and a CER of 5.0% for Tesseract on handwritten text represents a competitive result given the variability in handwriting styles, image quality, and domain-specific vocabulary. This is consistent with findings in the literature where handwritten medical text CER values typically range from 4% to over 20% depending on writing legibility and preprocessing quality. The 5.0% CER achieved here reflects the benefit of the adaptive routing strategy that first isolates handwritten images before OCR processing. These findings indicate that the choice of the OCR engine by the type of image is highly effective in enhancing recognition of the images as opposed to employing the use of one OCR model to all the inputs. Furthermore, as a preliminary illustrative example rather than a quantitatively validated result, the extracted text from digital prescription images was post-processed to flag candidate medicine names using keyword matching against a structured drug lexicon; this suggests, but does not yet demonstrate, that the pipeline could support downstream pharmacological information retrieval, and a quantitative evaluation of this step (e.g., precision/recall against annotated drug mentions) is left for future work. As a comparative baseline, GPT-4o (OpenAI, model gpt-4o-2024-05-13) was evaluated on the identical dataset under the same ground-truth reference. GPT-4o achieved a CER of 3.4% and a WER of 6.8%, outperforming the proposed pipeline (CER 4.1%, WER 7.6%) in aggregate error metrics. This performance advantage is attributable to GPT-4o's multimodal pretraining and contextual reasoning, which enable it to resolve handwriting ambiguities and low-quality image artifacts that challenge rule-based OCR engines. It is important to acknowledge this finding directly: a single general-purpose multimodal model achieves lower error rates with a simpler deployment architecture. The proposed pipeline's advantage lies in its transparency, modularity, and cost-efficiency. Unlike GPT-4o, which operates as a black-box API with per-call cost, the proposed system uses open-source components (GhostNetV2, PaddleOCR, Tesseract) that can be deployed locally, audited, fine-tuned, and operated without ongoing API costs. For resource-constrained clinical or pharmacy settings, this constitutes a practical trade-off. Notably, the suggested pipeline offers a viable trade-off between efficiency and accuracy in computations, whereas the extraction based on OpenAI serves as a powerful comparative benchmarking baseline. These results validate the idea that lightweight classification combined with specialized pre-trained OCR engines and LLM-based comparison can provide a robust and reliable system to digitize medical prescriptions in practice.

A comparison table that reports the computational complexity of GhostNetV2 alongside several widely used lightweight CNN architectures, including MobileNetV2, MobileNetV3, ShuffleNetV2, and EfficientNet-B0 is listed in Table 1. Table 1 highlight that GhostNetV2 attains classification accuracy, precision, recall, and F1-score essentially tied with the best-performing backbone, EfficientNet-B0 (both at 99.0% accuracy), while requiring substantially fewer parameters and FLOPs. GhostNetV2's advantage over the other evaluated backbones is therefore strictly a computational-efficiency advantage rather than a classification-accuracy advantage: it matches the top accuracy while using roughly a quarter of EfficientNet-B0's FLOPs (0.17 vs. 0.39 GFLOPs) and fewer parameters (3.9M vs. 5.3M), and it achieves markedly lower latency than every other backbone in Table 1. As with Table 2, the accuracy figures in Table 1 come from a single 80/20 split; Section 4.4.1 shows via 5-fold cross-validation that single-split accuracy for this dataset size can vary by several points, so the near-tie between GhostNetV2 and EfficientNet-B0 reported here should be read with that same caveat in mind pending a full cross-validated backbone comparison. Its moderate parameter count and low FLOPs make it an appropriate backbone for the proposed adaptive OCR routing framework. For reproducibility, all backbones in Table 1 (MobileNetV2, MobileNetV3-Small, ShuffleNetV2, EfficientNet-B0, and GhostNetV2) were trained from scratch under the identical protocol described in Section 4.3: the same 80/20 train/test split of the 508-image dataset, 50 epochs, the Adam optimizer with a learning rate of 1 × 10⁻^3^, a batch size of 32, and 512 × 512 input resolution, so that the reported accuracy, parameter count, FLOPs, and latency figures are directly comparable across models.

The routing module performs only binary classification to determine whether a prescription image is handwritten or digital before OCR execution as listed in Table 4. Therefore, minimizing computational complexity is more important than employing a large-capacity backbone. As shown in Table 4, GhostNetV2 provides the best trade-off between classification performance and computational efficiency, achieving 99.0% accuracy with only approximately 3.9 million parameters and 0.17 GFLOPs. Compared with EfficientNet-B0, GhostNetV2 requires substantially fewer floating-point operations while maintaining comparable classification performance, making it suitable for deployment in real-time pharmacy information systems and resource-constrained environments.

Since the proposed classifier serves only as a routing mechanism for selecting the appropriate OCR strategy, minimizing computational complexity is essential to reduce the overall inference time of the complete pipeline. GhostNetV2 achieves a favorable balance between classification accuracy and computational efficiency by generating intrinsic feature maps using inexpensive linear operations, thereby reducing redundant feature computation. Consequently, it provides an efficient backbone for real-time prescription-image classification without introducing significant computational overhead prior to OCR processing.

#### 4.4.2 Bilingual OCR configuration check

The English-only PaddleOCR configuration used above is a controllable experimental choice rather than an inherent limitation of the tool, since a substantial portion of the digital-class images contain Arabic-script text. To quantify the effect of this choice, we re-ran the digital branch with a bilingual (English + Arabic) PaddleOCR configuration and compared it against the original English-only configuration. PaddleOCR does not provide a single combined English + Arabic recognition model in the way Tesseract does; the bilingual configuration was therefore implemented by running PaddleOCR's English and Arabic recognizers independently over each image and merging their output region-by-region, keeping the higher-confidence recognition for each detected text box. Because the original manually-verified ground-truth transcriptions used elsewhere in this study were not available for this supplementary re-run, both configurations were scored against an automated silver-standard reference generated with Qwen2.5-VL (open-weights, run locally), with a self-consistency check flagging images with unstable transcriptions; these results should accordingly be read as a *relative* comparison between the two PaddleOCR configurations rather than a replacement for the human-verified 3.2% CER figure reported above.

## 5 Conclusions and future work

The current paper introduced a complete intelligent system of digitizing medical prescriptions automatically by incorporating all the practical issues related to the processing of heterogeneous prescription images that are exchanged via the web environment and social media. The proposed system, having a lightweight GhostNetV2 classifier as a front-end decision module, is able to classify between handwritten and digital prescriptions and route them to the most appropriate OCR engine. Wide-scale experimental research proved this adaptive design greatly enhances the accuracy and reliability of text extraction in comparison with single-OCR methods and proves the usefulness of routing using classification and specialized OCR model combination.

The findings showed that while GPT-4o achieves marginally lower error rates, the proposed pipeline offers competitive performance with substantially greater transparency, modularity, and deployability. The use of open-source, locally deployable components (GhostNetV2, PaddleOCR, Tesseract) makes the system auditable and cost-efficient, which are critical attributes in healthcare deployments where explainability and data privacy are paramount. In general, it can be stated that the suggested system is a scalable, modular, and cost-efficient approach to digitization of prescriptions in the real world.

### 5.1 Limitations

Two limitations of the evaluation protocol were identified during peer review; one has since been addressed with additional experiments, and one remains open. First, the classification metrics originally reported in Table 2 (99.0% accuracy) came from a single 80/20 train/test split of a relatively small, 508-image dataset, with only 41 handwritten images in the test split, raising the concern that single-split evaluation of this kind can be sensitive to the particular split drawn. We addressed this directly by running 5-fold stratified cross-validation over the full dataset (Section 4.4.1, Table 3), retraining GhostNetV2 from scratch on each fold under the identical protocol. The result confirms the concern: the cross-validated accuracy is 93.1% ± 3.0% (mean ± SD across folds), materially lower than the original single-split figure, showing that the 99.0% estimate was optimistic. We now report 93.1% ± 3.0% as the representative classification performance of GhostNetV2 and treat the single-split Table 2 figures as specific to the confusion-matrix/ROC analysis computed on that split rather than as the paper's headline accuracy claim. The same caveat applies to the single-split backbone comparison in Table 1, which has not yet been re-run with cross-validation; doing so for all five backbones is left for future work. Second, PaddleOCR was run in an English-only configuration although the dataset contains Arabic-script prescriptions; this remains a controllable configuration choice rather than an inherent constraint, and re-running the digital branch with a bilingual (English + Arabic) PaddleOCR configuration to report both sets of results is still an open, actionable next step that has not yet been carried out. The CER/WER figures reported in Section 4.4 should accordingly still be read as an English-only estimate of digital-branch OCR performance.

A current direction of work, in future applications, is to broaden the classifier to more than binary classification and that to other prescription characteristics like language, a complex layout, image quality or content mixed with hand-written or printed content. This would enable finer grained OCR routing and also minimize the recognition errors. Besides this, more robustness in low-quality pictures due to low-light conditions or compression artifacts typical of messaging apps can be enhanced with the integration of advanced image enhancement and super-resolution algorithms before classification.

The other promising future direction is the incorporation of domain-specific large language models to conduct semantic post-processing, e.g., medication name normalization, dosage check, and drug-drug interaction checks. It would be beneficial to further test the usefulness of the system by extending it to allow multilingual prescriptions and implementing it in a clinical or pharmacy setting in a real-time environment. Lastly, the human-in-the-loop feedbacks may be used to constantly enhance the performance of the model, which would provide long-term flexibility and stability in the dynamic healthcare environment.

## Data Availability

The raw data supporting the conclusions of this article will be made available by the authors, without undue reservation.
